# Fenestrated Transcatheter Arterialization of the Deep Veins: Protecting the Peroneal While Covering the Tibioperoneal Trunk

**DOI:** 10.1016/j.jaccas.2025.105208

**Published:** 2025-09-10

**Authors:** Brenden S. Ingraham, Arashk Motiei

**Affiliations:** Department of Cardiovascular Diseases, Mayo Clinic, Rochester, Minnesota, USA

**Keywords:** peripheral circulation, peripheral vascular disease, vascular disease

## Abstract

**Objective:**

We describe a novel approach to transcatheter arterialization of the deep veins (TADV) in a patient with chronic limb-threatening ischemia using the LimFlow kit and a Culotte 2-stent bifurcation technique to maintain peroneal flow while deploying a covered stent graft into the tibioperoneal trunk and posterior tibial vein when there is no tibial artery suitable for the TADV inflow.

**Key Steps:**

(1) Antegrade access of the superficial femoral artery and ipsilateral lateral plantar vein, (2) crossover wire from the artery into the vein, (3) place the LimFlow stent graft connecting the artery to the vein, (4) fenestrate the stent graft to allow bifurcation stenting into the peroneal artery to preserve native peroneal flow, and (5) optimize the inflow and outflow from the graft.

**Potential Pitfalls:**

Plantar venous access, compromising native arterial flow, and graft inflow/outflow issues require careful consideration during and after the TADV procedure.

**Take-Home Message:**

Alternative inflow TADV is feasible to maintain native arterial perfusion while the TADV circuit matures.

## Graphical Abstract

**Figure:**
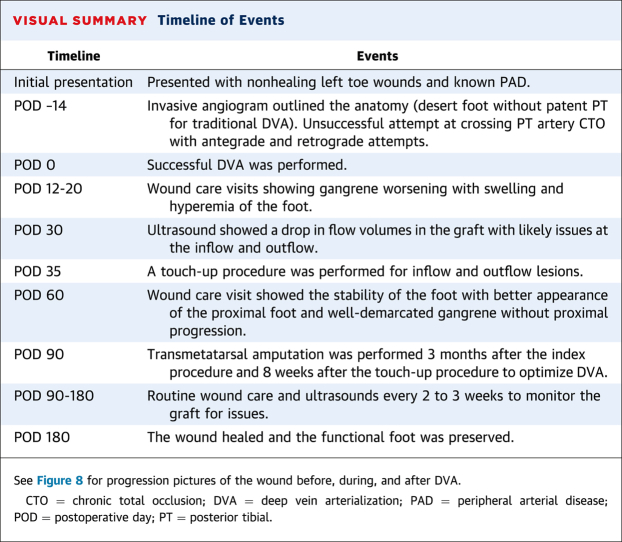

Transcatheter arterialization of the deep veins (TADV, also called deep vein arterialization or DVA) is an endovascular procedure for “no-option” chronic limb-threatening ischemia (CLTI), meaning there is no adequate distal arterial target for endovascular or surgical therapy. This subset of CLTI has patent above-the-knee vasculature but diseased tibial and pedal arteries with nonhealing wounds. More than 50% will progress to major amputation at 6 months.^1^^,^^2^ TADV uses healthier veins as conduits to deliver oxygenated blood to the ischemic foot to avoid or minimize tissue loss and amputation.^3^^,^^4^ LimFlow (Inari Medical) is the only FDA-approved TADV device. It streamlines the procedure with a toolkit designed for TADV and the unique shapes, sizes, and flow dynamics of TADV. The prospective, multicenter, safety, and efficacy PROMISE II trial of patients with no-option CLTI noted a 99% technical success rate, 76% functional limb preservation at 6 months, and 76% healing of chronic wounds at 6 months.^1^ LimFlow includes the ARC device (LimFlow) to cross from the arterial inflow into the vein, the V-Ceiver (LimFlow) grasps the wire to externalize through the foot, and the Vector valvulotome (LimFlow) disrupts the venous valves to prepare the vein for the covered stent graft. The stent graft has cylindrical 5.5-mm segments distally for the veins and a conical 5.5-mm tapering to a 3.5-mm graft that is deployed at the inflow segment. A crucial principle of TADV is the understanding that the high flow into the large graft steals flow from native arteries and can worsen limb ischemia during the 6 to 9 weeks it takes for the TADV circuit to mature. Maturation or neovascularization is when the veins develop toward the distal tissues to provide arterial-like small vessels to deliver oxygen to the at-risk tissues.^5^ The covered stent must not cover native vessels or supportive collaterals or TADV can cause acute worsening and lead to limb loss before the TADV circuit matures. We present a novel case of arterial inflow from the tibioperoneal (TP) trunk and fenestration of the stent graft to preserve flow into the native peroneal artery.

**Take-Home Messages**

• TADV requires an arterial inflow, and novel approaches—such as using the tibioperoneal trunk and fenestration into the peroneal artery in this case—to create the inflow may be necessary.
• This technique maintained native arterial perfusion while the TADV circuit matured, which ultimately saved the patient from a below-the-knee amputation and healed the transmetatarsal amputation wound.

## Case Summary

**Figure 1:**
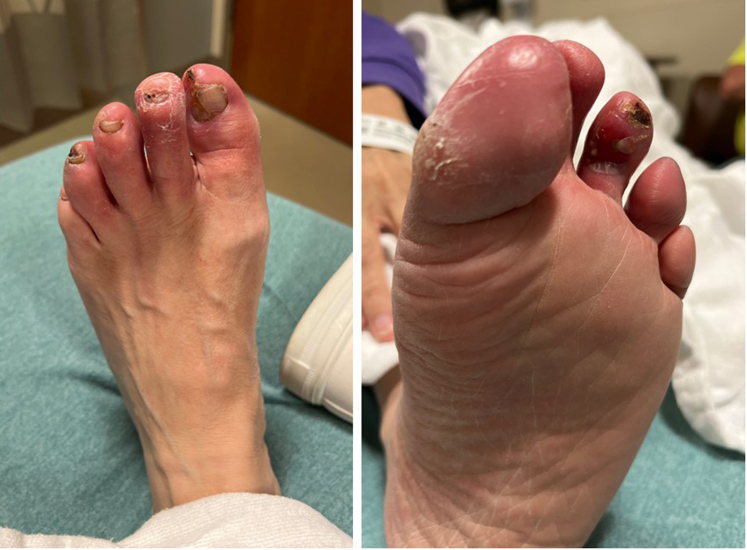
Left Toe Ulcers—Initial Presentation

**Figure 2:**
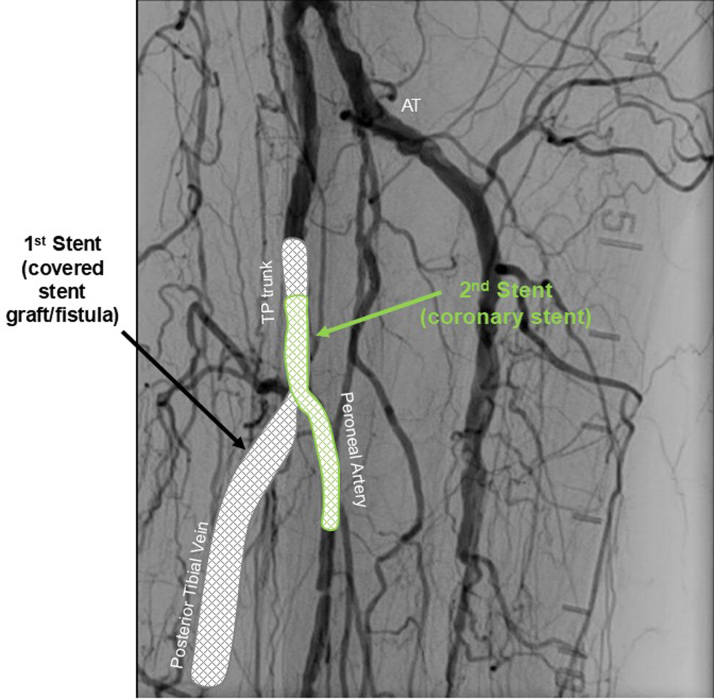
Angiogram With Stent Graft Fenestration and Culotte Stenting

A 63-year-old woman with a history notable for peripheral arterial disease with right below-the-knee amputation 1 year prior, end-stage renal disease with remote failed transplant and chronic dialysis, hypertension, and hyperlipidemia presented with nonhealing left toe ulcers (Figure 1) and rest pain. The ankle brachial index indicated noncompressible vessels with a reduced toe brachial index of 0.25 and severely reduced transcutaneous oxygen measurement at 24 mm Hg. The computed tomography angiogram showed no flow-limiting lesions above the knee and indeterminate tibial vessels. She was taken for invasive angiography (5-F left superficial femoral artery [SFA] access). Multifocal occlusions of the left anterior tibial artery were noted; distal vessels and dorsalis pedis were occluded as well. The peroneal artery was patent but had focal calcific stenoses. The posterior tibial (PT) artery was completely occluded in the proximal segment and reconstituted in the distal segment via peroneal collaterals. The common plantar artery and distal plantar vessels were patent but were underfilled. The pedal arch was occluded. The distal forefoot exhibited diffuse small-vessel disease. The bulky calcified anterior tibial and PT lesions were unable to be crossed using antegrade and retrograde techniques with heavy weight chronic total occlusion wires. She was diagnosed with no-option CLTI and arranged for TADV using the LimFlow device with plans to fenestrate the covered stent graft to preserve peroneal flow (Figure 2).

## Procedural Steps

**Figure 3:**
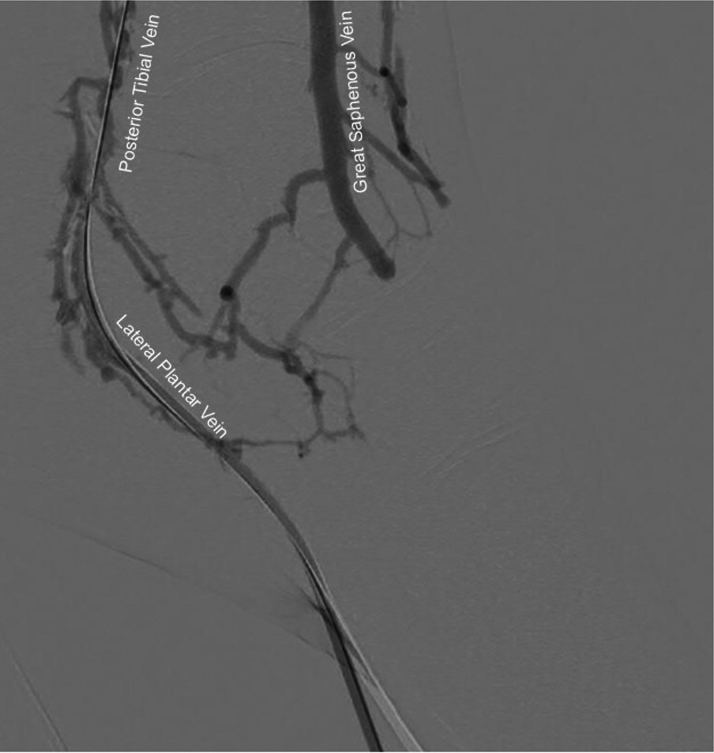
Pedal Venogram

**Figure 4:**
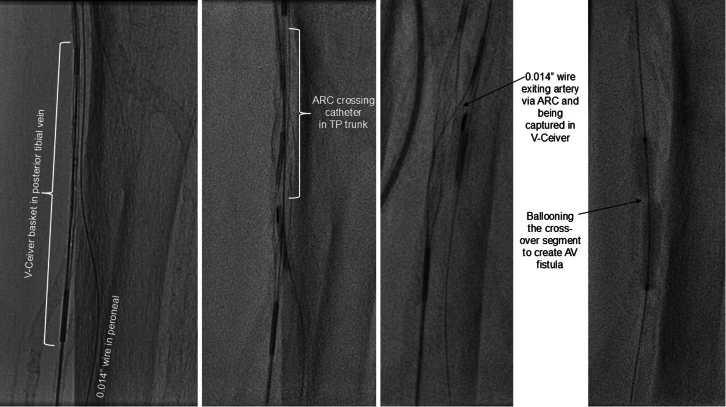
Creating an Arteriovenous Fistula From the TP Trunk Into the Posterior Tibial Vein TP = tibioperoneal.

**Figure 5:**
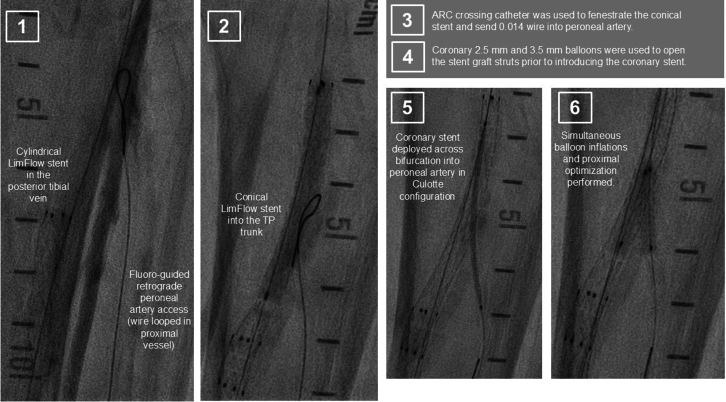
Fenestration of the Stent Graft and Bifurcation Stenting

**Figure 6:**
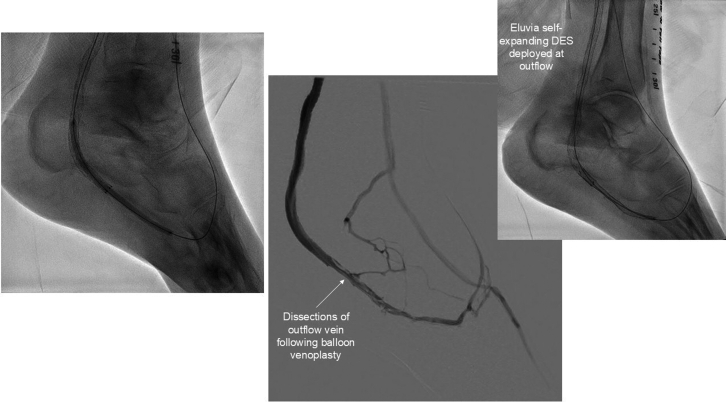
Venoplasty of Outflow and Arch

**Figure 7:**
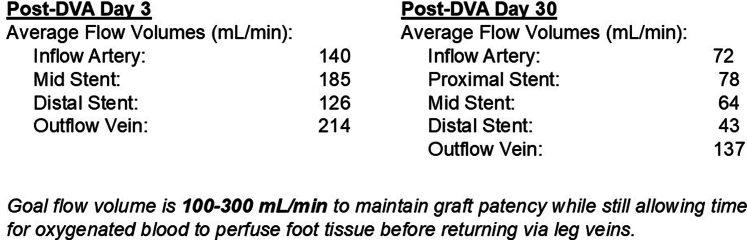
DVA Ultrasound Postprocedure: 3 Days and 30 Days DVA = deep vein arterialization.

**Figure 8:**
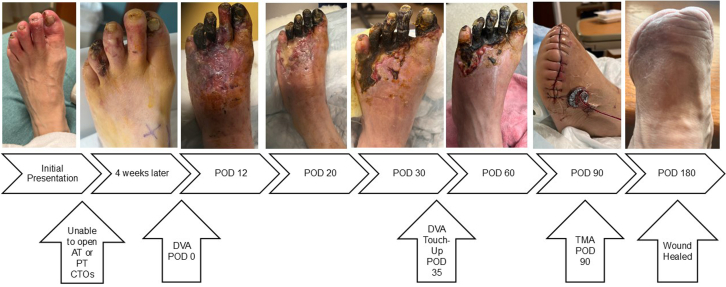
Wound Progression Pictures AT = anterior tibial; CTO = chronic total occlusion; POD = post-operative day; PT = posterior tibial; TMA = transmetatarsal amputation.

• The patient was placed under general anesthesia given the prolonged procedure time and preference for obtaining venous access without lidocaine, which obscures ultrasound imaging.
• Ultrasound-guided micropuncture antegrade SFA access and lateral plantar vein access were obtained. Lateral plantar vein access (without lidocaine) was obtained initially with a 21-gauge × 7 cm needle, a Nitrex 0.018-inch wire (Medtronic), and a stiff micropuncture sheath. The Nitrex wire was exchanged for a Command ST 0.018-inch wire (Abbott) from the lateral plantar vein into the popliteal vein. The micropuncture sheath was replaced with a 4/5-F Glidesheath Slender (Terumo Medical Corporation). The micropuncture needle and Nitrex wire were used to access the very proximal SFA (lidocaine given) with a micropuncture sheath. The STORQ wire (Cordis) was used to navigate down the SFA and placed in the popliteal artery for support. A skin nick was made to facilitate vascular closure at case completion; a 7-F short sheath was placed in the left SFA to dilate it, and then, it was swapped for a 7-F × 45 cm Destination sheath (Terumo Medical Corporation) with the distal tip placed in the popliteal artery.
• Command ES 0.014-inch wires (Abbott) were placed in each vessel (the LimFlow kit requires 0.014-inch wires).
• An angiogram was performed from the popliteal artery (Figure 2), and a pedal venogram was performed through the lateral plantar vein sheath with a tourniquet on the ankle to enhance the venogram by restricting venous return (Figure 3).
• An ARC device was used from above to cross from the TP trunk into the PT vein (V-Ceiver captures the venous wire to externalize), which allows through-and-through wiring from the proximal artery inflow into the distal venous outflow.
• Balloon angioplasty (4-mm coronary balloon) of the fistula crossover site was performed to open connection between the artery and the vein, and then the PT vein was prepared using the Vector valvulotome and balloon venoplasty (5 × 220 mm).
• LimFlow cylindrical covered stent grafts were deployed in the PT vein (5.5 × 200 mm and 5.5 × 150 mm).
• Fluoroscopic retrograde access of the peroneal artery leaving the marker wire (micropuncture sheath and Command ST 0.018-inch wire) was obtained.
• A conical stent graft was deployed across the arteriovenous crossover (5.5-3.5 tapered × 60 mm) to transition the vein to an inflow artery.
• The ARC device was used to perforate the covered stent and wire into the peroneal artery; 2.5- and 3.5-mm coronary balloons were used sequentially to predilate the fenestration to prepare for the side branch stent (Figure 4).
• A 3.5 × 38 mm Synergy coronary drug-eluting stent (DES) was deployed into the peroneal artery, extending several millimeters back into the TP trunk.
• The main branch (stent graft in the PT vein) was rewired, and kissing balloon inflation of the bifurcation (3.5- and 3.0-mm balloons in the fistula and peroneal artery, respectively) and proximal optimization of the proximal stent were performed using a 4-mm noncompliant balloon (Figure 5).
• Angioplasty of the peroneal artery was performed using a tapered 2.5 to 3.0 mm × 210 mm balloon, followed by prolonged balloon inflation (2.5 × 30 mm) of the distal peroneal access site for hemostasis.
• The venous arch was wired; balloon 3-mm venoplasty of the arch was performed to improve outflow into the forefoot veins, and 4-mm venoplasty of the lateral plantar vein was performed after removing the plantar sheath with a 4-minute inflation to assist hemostasis after pedal sheath removal (Figure 6).
• Significant dissection occurred at the distal stent graft edge; a 6 mm × 60 mm Eluvia (Boston Scientific Corporation) self-expanding DES was deployed to cover the dissections.
• The femoral site was closed using a suture-based closure device (Prostyle Perclose; Abbott).
• Monitoring ultrasound at 1 month showed a diminished flow volume rate at the proximal stent edge, which severely reduced at the distal stent (Figure 7).
• A repeat angiogram showed inflow and outflow lesions; an Eluvia DES was deployed to cover inflow lesions, and venoplasty was performed distally to optimize outflow.
• Transmetatarsal amputation (TMA) was performed 8 weeks later.
• Two additional procedures—balloon dilations of the graft and outflow vein—were necessary to assist graft patency. The peroneal and collaterals provided significant native arterialization of the forefoot apparent on the final angiogram.
• The wound completely healed 3.5 months after TMA (Figure 8).

## Potential Pitfalls

Accessing the lateral plantar vein is often the most challenging part of the procedure, especially in patients with small, diseased plantar veins. Keeping the patient warm and sedated is important to combat spasm. Lidocaine can also severely diminish visibility on ultrasound. We favor general anesthesia to allow for deep sedation, immobility, and venous access without lidocaine. When there is single vessel runoff into the lower leg, preserving the native arterial flow to the foot is important. Steal from the graft or losing vessels (including collaterals) can lead to acute worsening of the ischemic foot and may result in the need for amputation before the TADV circuit has time to mature. Regular ultrasounds every 2 to 4 weeks are important to monitor graft flow and patency to ensure prompt efforts at revascularization and optimization as needed. Careful patient selection and setting the expectation of frequent clinic visits, ultrasounds, and invasive procedures is often required to avoid major amputation.

**Equipment List:** 

• Ultrasound with a vascular probe • 21-gauge × 7 cm micropuncture access needle • Nitrex 0.018-inch wire (Medtronic) • STORQ 0.035-inch wire (Cordis) • 7-F × 45 cm Destination Sheath (Terumo Medical Corporation) • 4/5-F Glidesheath Slender (Terumo Medical Corporation) • Command ES 0.014-inch wire × 3 (Abbott) • Command ST 0.018-inch wire (Abbott) • LimFlow TADV System with ARC, V-Ceiver, Vector valvulotome, and covered stent grafts—sizes 5.5 × 200 mm, 5.5 × 150 mm, 5.5-3.5 tapered × 60 mm (Inari Medical) • 2.5, 3.0, 3.5, 4.0 coronary balloons (approximately 30 mm length) • 5 × 220 mm peripheral balloon • 2.5-3.0 mm × 210 mm peripheral tapered balloon • 3.5 × 38 mm Synergy coronary DES (Boston Scientific Corporation) • Eluvia 6 mm × 60 mm self-expanding DES (Boston Scientific Corporation) • Prostyle Perclose (Abbott)

**Visual Summary:** Timeline of Events

Timeline	Events
Initial presentation	Presented with nonhealing left toe wounds and known PAD.
POD –14	Invasive angiogram outlined the anatomy (desert foot without patent PT for traditional DVA). Unsuccessful attempt at crossing PT artery CTO with antegrade and retrograde attempts.
POD 0	Successful DVA was performed.
POD 12-20	Wound care visits showing gangrene worsening with swelling and hyperemia of the foot.
POD 30	Ultrasound showed a drop in flow volumes in the graft with likely issues at the inflow and outflow.
POD 35	A touch-up procedure was performed for inflow and outflow lesions.
POD 60	Wound care visit showed the stability of the foot with better appearance of the proximal foot and well-demarcated gangrene without proximal progression.
POD 90	Transmetatarsal amputation was performed 3 months after the index procedure and 8 weeks after the touch-up procedure to optimize DVA.
POD 90-180	Routine wound care and ultrasounds every 2 to 3 weeks to monitor the graft for issues.
POD 180	The wound healed and the functional foot was preserved.

See Figure 8 for progression pictures of the wound before, during, and after DVA.

CTO = chronic total occlusion; DVA = deep vein arterialization; PAD = peripheral arterial disease; POD = postoperative day; PT = posterior tibial.

## Conclusions

Fenestration of the TADV covered stent graft is feasible and effective to maintain native arterial flow when the traditional PT inflow segment is occluded and cannot be used for TADV. This novel approach to TADV allowed our patient with no-option CLTI to proceed with TMA, heal the surgical wound, and preserve a functional foot.

## Funding Support and Author Disclosures

The authors have reported that they have no relationships relevant to the contents of this paper to disclose.

## Appendix

Video 1 Initial Angiogram

Video 2 Completion Angiogram After Deep Vein Arterialization

Video 3 Inflow and Outflow Lesions on a 30-Day Angiogram

## References

[1] Shishehbor M.H., Powell R.J., Montero-Baker M.F. (2023). Transcatheter arterialization of deep veins in chronic limb-threatening ischemia. N Engl J Med.

[2] Duff S., Mafilios M.S., Bhounsule P., Hasegawa J.T. (2019). The burden of critical limb ischemia: a review of recent literature. Vasc Health Risk Manag.

[3] Gornik H.L., Aronow H.D., Goodney P.P., Writing Committee Members (2024). 2024 ACC/AHA/AACVPR/APMA/ABC/SCAI/SVM/SVN/SVS/SIR/VESS guideline for the management of lower extremity peripheral artery disease: a report of the American College of Cardiology/American Heart Association joint committee on clinical practice guidelines. J Am Coll Cardiol.

[4] Clair D.G., Mustapha J.A., Shishehbor M.H. (2021). Promise I: early feasibility study of the LimFlow system for percutaneous deep vein arterialization in no-option chronic limb-threatening ischemia: 12-month results. J Vasc Surg.

[5] Rice J.R., Chatman B.C., Genovese E.A. (2025). Percutaneous approach to deep vein arterialization for the “no-option” chronic limb-threatening ischemia patient. Semin Vasc Surg.

